# Suicidal Ideation among the Chinese Elderly and Its Correlates: A Comparison between the Rural and Urban Populations

**DOI:** 10.3390/ijerph15030422

**Published:** 2018-02-28

**Authors:** Jianwen Wei, Jie Zhang, Yuping Deng, Long Sun, Ping Guo

**Affiliations:** 1 School of Sociology, Beijing Normal University, Beijing 100875, China; stanwjw@163.com; 2 Central University of Finance and Economics School of Social Development, Beijing 100081, China; dengyuping900522@sina.com; 3 State University of New York Buffalo State Department of Sociology, Buffalo, NY 14222, USA; 4 Shandong University School of Public Health Center for Suicide Prevention Research, Jinan 250012, China; longsun.sdu@gmail.com; 5 China Research Center on Aging, Beijing 100054, China; pingguo89@hotmail.com

**Keywords:** the Chinese elderly, depression, suicidal ideation, risk factors, urban-rural comparison

## Abstract

Background: As China is going through a profound aging process, the mental health of the elderly is becoming an issue. As in many other societies, the elderly in China is a population at high risk of suicide; Methods: Data for the study were taken from the Sample Survey of the Aged Population in Urban/Rural China (SSAPUR) accomplished in 2010 by the China Ministry of Civil Affairs. The valid sample for this study was composed of 18,683 individuals, including 9416 urban residents and 9267 rural residents both aged 60 or more years; Results: Logistic regression analyses showed that household income and expenditure, the number of children, chronic diseases, disability of daily living, depression, the frequency of visiting neighbors and having friends or relatives who can help or not had remarkable effects on the suicidal ideation among urban and rural old people. Gender, education, political affiliation, marital status and self-rated health status did not work on the dependent variable. However, some risk factors for suicidal ideation among the Chinese elderly were different between rural and urban regions; Conclusions: We should take different measures when facing the different groups of the elderly.

## 1. Introduction

Aging is an increasing problem in China today. According to the data of the Sixth National Population Census in 2010, the number of people aged above 60 years was 177,648,705, accounting for 13.26% of the total population. It had increased by 2.93% compared with the proportion in 2000 [1]. In contrast to the developed countries, which became rich before their populations became old, the population of China is going through a profound aging process before it becomes wealthy. Because China has not fully prepared for the aging, the elderly are encountering varieties of challenges, such as suicide [2].

China had the third highest rate of suicide among the elderly in the world and the over-65 age group had the highest rate of completed suicide in China, which reached 44.3–200 per 100,000 [3]. From this perspective, we have enough reasons to believe that the prevalence of suicidal ideation among the Chinese elderly was high, too. As an indicator of potential suicidal attempts and behaviors, suicidal ideation should be well-studied, as well as its influencing factors, which can help us to prevent and handle the fatal results of suicide. In this paper, the first question we want to discuss is the prevalence of suicidal ideation among the Chinese elderly and its correlates.

The urban-rural dual system is one of social structural characteristics of China and there exist population differences between cities and countries. Many problems in China must be considered and solved in rural/urban specific ways. When it comes to the suicide rate, the rural elderly contribute much more than the urban elderly. To go much further, the suicide rate among the rural elderly was approximately three to five times higher than that among the urban elderly [3,4]. Hence, the second question we want to explore in this paper is the differences of the status quo and correlates of suicidal ideation between the urban and rural elderly.

Suicidal ideation in later life is best understood as a multidimensional event. Based on the literature from the West, we have summarized eight correlates of suicidal ideation for the elderly, namely, demographic characteristics, socio-economic status, marital status, physical health, mental health, influence of major events, religious belief and social interaction.

First, demographic characteristics were the basic information of each one, which would inevitably have some effects on people’s suicidal ideation to a certain degree, such as female, old age, etc. [5]. Socio-economic status was another indispensable aspect and many scholars have researched the relationship between socio-economic status and suicidal ideation [6]. Family factors provided the most relevant social context for people, so marital status was one of the evident indicators showing the family background. A variety of studies have testified that the unmarried elderly had a higher prevalence of suicidal ideation than the married. Besides the conjugal bond, the parenthood relation cannot be ignored either. Physical condition and the following functional impairment would increase the suicide rates when people were in later life [7] and people who have suicidal intent were frequently living in poor physical health [8]. Besides physical health, psychological condition was also powerful for predicting suicidal ideation and the well-being of psychological states, which can have negative effects on suicidal ideation [9,10,11]. Social interaction and social participation were also considered as factors that affect suicidal ideation. Social connectedness was negatively associated with suicidal intent [12,13]. What’s more, the influence of major events would arouse more or less life stress and appear to be an important factor to explain self-harm behavior [14,15]. Previous research had come to a similar conclusion: religious belief had a relation to mental states and further affects the suicidal ideation of people but the its role was indefinite [16].

The literature on elderly suicidal ideation was limited for the Chinese populations. This study contributes to the literature by adding the significant correlates of Chinese elderly suicidal ideation with a comparison between the rural and urban populations.

## 2. Method

### 2.1. Data Sources and Samples

The data used in this study were taken from the Sample Survey of the Aged Population in Urban/Rural China (SSAPUR), which were conducted by China Research Center on Aging (CRCA) on December, 2010 [17].

The Probability Proportional to Size Sampling was used in the sampling process. First, we randomly selected 20 provinces from thirty-one provinces (including municipalities and autonomous regions) in mainland China. Secondly, we randomly selected four cities (in urban areas) or four counties (in rural areas) from each province. Thirdly, we randomly selected 16 sub-districts from each city and 16 towns from each county. Fourthly, we chose 50 communities from each sub-distribute and 50 villages from each town. If there were not 50 communities or villages, we chose all of them. Finally, we selected 10 families that included a person aged above 60 years old from each community or village. One old person would be interviewed from each of the families. Therefore, we obtained 19,986 samples attending this household survey in total. For detailed information of the data collection design and administration, please refer to an earlier publication in Chinese [17].

The data were obtained by questionnaire survey, which was self-administered or assisted by survey staff if needed. The survey place was mostly at the respondent’s home. A total number of 19,986 questionnaires were collected. After the data cleaning, the number of valid cases for this research was 18,693, accounting for 93.53% of the collected cases.

### 2.2. Measurement of Variables

#### 2.2.1. Dependent Variable

In SSAPUR, there were three items to reflect the state of suicidal ideation, including the prevalence of suicidal ideation in the past 5 years, 1 year and 1 month. The question for 1 year ideation was “Have you ever seriously consider killing yourself in last 1 year?” Among the 18,237 people who answered the first question, 878 reported suicidal ideation, accounting for 4.81%. About the same number of respondents answered the second question, with 707 reporting suicidal ideation, accounting for 3.88%. A total number of 18,683 elderly responded to the third question and 518 of them reported suicidal ideation, accounting for 2.77%. Considering the accuracy of suicidal ideation among the elderly, we chose the item for suicidal ideation in the past one month as the dependent variable. This variable is of binary-class, Yes = 1 and No = 0.

#### 2.2.2. Independent Variables

In the dimension of **demographic characteristics**, Gender and Age were basic demographic variables. Gender was a binary variable and Male = 1 and Female = 0. The age was a continuous variable.

**Socio-economic status** was measured by education level and financial situation. The *education level* was graded into illiteracy, primary school (including *Sishu*, a kind of old-style private school in China) and junior high school, which was ordered from low to high. *Financial situatio*n was graded into sufficient, relatively sufficient, roughly enough, relatively insufficient and insufficient, which was ordered from rich to poor.

**CCP membership** was judged by a question that “whether he/she was a Chinese Communist Party (CCP) member”, and Yes = 1 and No = 0.

**Family factors** were reacted by marital status and the number of children. *Marital status* was divided into married and not married and Yes = 1 and No = 0. *The number of children* means the current quantity of children, not including the children who had passed by. This variable was continuous.

**Physical condition** was evaluated by chronic diseases, self-evaluation of the health and disability of daily living. Whether have *chronic diseases* was a binary variable and Yes = 1 and No = 0. *Self-evaluation of the health* was an ordinal variable and classified into very unhealthy, unhealthy, average, healthy and very healthy. *Disability of daily living* was measured by the ADL Scale, which had investigated their ability on eating, dressing, toileting, getting in and out of bed, bathing and walking indoors. The answers of every question were divided into “can do it easily”, “can do it with some difficulties” and “absolutely cannot do it”. Three answers were separately marked into 1, 2 and 3. We had computed the average marks of the six questions above and the higher the score was, the higher the degree of disability was.

**Mental condition** was reflected by level of *depression* that was measured by the Geriatric Depression Scale-15 (GDS-15). This scale was comprised of 15 questions which investigated some feelings and behaviors in the past one week. This scale had been widely used in China. The reliability and validity were also testified in previous studies [18,19,20]. The answers of each question were divided into yes or no and Yes = 1 and No = 0. We had added up all the questions and got the total score; the higher the score is, the more severe the depression is.

**Social interaction** was estimated from four aspects: whether they participated in social activities, whether they have friends or relatives who can chat with them deeply, whether they have friends or relatives who can give them a hand when they need help and whether they visit neighbors. The former three variables were binary and Yes = 1 and No = 0; the answers of the last one were divided into frequently, occasionally and never.

**Influence of major events** was another factor that may function, measured by whether the respondent has encountered some important events which made great effects on their family in the recent one year, such as natural hazards, diseases, accidents, lawsuits, dispute and land acquisition and Yes = 1 and No = 0.

**Religious belief** was a binary variable. If the respondent believes in any particular religion, the answer was marked as 1, otherwise we marked it with 0.

### 2.3. Statistical Method

Data were managed and analyzed with SPSS 17.0. First, we used descriptive statistics to assess the distribution of samples. Then we showed the prevalence of suicidal ideation according to each independent variable. T-tests or Chi-square tests were used to compare the difference on categorical and continuous variables across groups. Further, because the suicidal ideation was a binary variable, so we used the logistic regression to verify the association between independent variables and the dependent variables. We built three models to illustrate the results of the overall, the urban and the rural elderly respectively.

## 3. Results

### 3.1. Basic Information of Samples

The sample description and distribution are shown in Table 1. The ages of the samples ranged from 60 to 103 and the average was 72.30.

### 3.2. Prevalence of Suicidal Ideation

**Figure 1:**
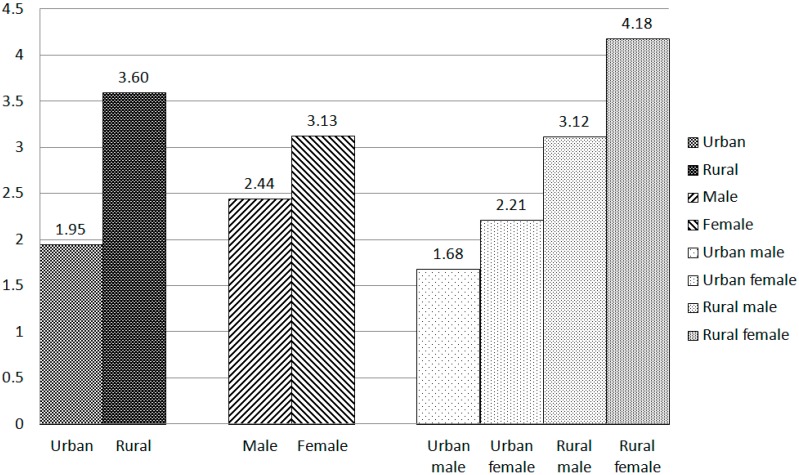
Prevalence of suicidal ideation among Urban and rural elderly (%).

The prevalence of suicidal ideation among the Chinese Elderly was revealed in Table 2. The prevalence of Suicidal ideation among the overall elderly was 2.77%. As Figure 1 showed, the female elderly in rural areas had the highest prevalence of suicidal ideation, up to 4.18%, while the male elderly in urban areas had the lowest prevalence of suicidal ideation, which was only 1.68%. The suicidal ideation was statistically significant between rural and urban regions (*χ*^2^ = 41.495, *p* < 0.001).

### 3.3. Logistic Analysis about the Suicide Ideation in Chinese Elderly

In order to discuss the explanatory power of the factors above, we put all the independent variables into the regression model. Specific information is shown in Table 3. According to the results of the logistic regression, gender, education level, political affiliation, marital status and self-rated health status were not statistically significantly associated with suicidal ideation but several variables did. What is more, the factors were different among urban and rural elderly.

## 4. Conclusions 

### 4.1. Main Findings of This Study

We found that age, social economic status, family factors, physical health, mental health, living ability, social interaction and religious belief did have effects on the prevalence of suicidal ideation. Household income and expenditure, the number of children, chronic diseases, disability in daily living, depression and social interaction had influence on both the urban and rural elderly. Better conditions of household income and expenditure will decrease the level of suicidal ideation. The elderly who have more children tend to have a higher level of suicidal ideation than those who have fewer or no children, which is especially true of the rural elderly. This can be explained with the Strain Theory of Suicide and mental disorder [21,22]. In rural China today with the fast growing economy, the elderly remain traditional in social values while the youth have started move away from the traditional Confucianism, which may create psychological strains for the elderly due to the discrepancy between their expectation of their children’s filial piety and the reality. Healthier old people and the elderly who can manage their daily living are less likely to think about suicide [8,23]. The level of depression is also a conspicuous factor [24].

However, several variables influencing the prevalence of suicidal ideation among rural and urban elderly were different. One of the important reasons is the large social environmental gap between rural and urban regions in China [25]. Among the urban elderly, age had a positive effect on the prevalence of suicidal ideation [26]. Among the rural elderly, religious belief had a positive effect on the prevalence of suicidal ideation, which has been identified in many previous studies in China [27].

### 4.2. What Is Already Known on This Topic and What This Study Has Added

Elderly populations are usually at a higher risk of suicide and mental problems in most societies, which is consistent with our results in the urban region [28,29]. We have explored in this study the influencing factors on the suicidal ideation among the Chinese elderly and found some risk factors especially in Chinese culture and certain differences between rural and urban elderly. These findings can be the basis for our future study as well as for policy-making in the efforts to reduce suicide among the elderly in China. In general, the prevalence of suicidal ideation in China might be a reflection of the social and economic transformation in the past few decades in the developing country. For example, the fast-growing economy brings about a cultural change in China. As society becomes industrialized from farming environments, modernized from traditional ideology and urbanized from a rural life style, younger generations are more likely to give up the traditional life and traditional social values than the elderly, while the latter still uphold traditional values such as Confucianism. One of the major teachings of Confucianism is filial piety, the veneration of the elderly by the children. While the Chinese elderly still hold the traditional values as important and as their expectation for their children, the younger generation are far more modernized than their parents and grandparents. This discrepancy between the elderly’s traditional aspirations and their children’s beliefs and behaviors can create some type of psychological strains among the elderly, which may make them feel frustrated, helpless, angered, or suicidal [21].

### 4.3. Limitations of This Study

Due to the limitations of the data and the research design, there may still be some important factors that were not considered in the relationship to Chinese elderly suicidal ideation. Besides, as the cultural differences in the China and Western countries, many correlates which have been supported in previous studies were not found in the current study [30]. We need to do further cross-cultural research on elderly psychopathologies and suicidal ideation.

## Figures and Tables

**Table 1:** Description of the Sample Characteristics.

	Frequency (%)/Mean (SD)		
Variable	Overall	Urban	Rural
***N***	18,683 (100.00)	9416 (50.40)	9267 (49.60)
**Gender**			
Male	9608 (51.43)	4573 (48.57)	5035 (54.33)
Female	9075 (48.57)	4843 (51.43)	4232 (45.67)
**Age**	72.30 (7.457)	72.33 (7.228)	72.27 (7.684)
**Education level**			
Illiteracy	5413 (28.97)	1435 (15.24)	3978 (42.93)
Primary school	7260 (38.86)	3141 (33.36)	4119 (44.45)
Junior high school or above	6010 (32.17)	4840 (51.40)	1170 (12.63)
**CCP membership**			
Yes	4163 (22.28)	3028 (32.16)	1135 (12.25)
No	14,520 (77.72)	6388 (67.84)	8132 (87.75)
**Financial situation**			
Sufficient	215 (1.15)	123 (1.31)	92 (0.99)
Relatively sufficient	2152 (11.52)	1277 (13.56)	875 (9.44)
Roughly enough	10,762 (57.60)	6006 (63.79)	4756 (51.32)
Relatively insufficient	4338 (23.22)	1577 (16.75)	2761 (29.79)
Insufficient	1216 (6.51)	433 (4.60)	783 (8.45)
**Marital status**			
In marriage	12,262 (65.63)	6520 (69.24)	5742 (61.96)
Not in marriage	6421 (34.37)	2896 (30.76)	3525 (38.04)
**Number of children**	3.21 (1.648)	2.85 (1.45)	3.58 (1.75)
**Chronic diseases**			
Yes	14,400 (77.08)	7721 (82.00)	6679 (72.07)
No	4283 (22.92)	1695 (18.00)	2588 (27.93)
**Self-rated health status**			
Very unhealthy	973 (5.21)	375 (3.98)	598 (6.45)
Unhealthy	3563 (19.07)	1434 (15.23)	2129 (22.97)
Average	9810 (52.51)	5241 (55.66)	4569 (49.30)
Healthy	3643 (19.50)	2013 (21.38)	1630 (17.59)
Very healthy	694 (3.71)	353 (3.75)	341 (3.68)
**Disability of daily living**	1.33 (0.440)	1.28 (0.420)	1.37 (0.460)
**Depression**	4.90 (3.400)	4.04 (3.120)	5.78 (3.450)
**Visiting neighbors**			
Frequently	5044 (26.00)	1912 (20.31)	3132 (33.80)
Occasionally	8765 (46.91)	4346 (46.16)	4419 (47.69)
Never	4874 (26.09)	3158 (33.54)	1716 (18.52)
**People who can help you**			
Yes	16,873 (90.31)	8441 (89.65)	8432 (90.99)
No	1810 (9.69)	975 (10.35)	835 (9.01)
**Negative life events**			
Yes	6863 (36.73)	3014 (32.01)	3849 (41.53)
No	11,820 (63.27)	6402 (67.99)	5418 (58.47)
**Religious belief**			
Yes	2640 (14.13)	1412 (15.00)	1228 (13.25)
No	16,043 (85.87)	8004 (85.00)	8039 (86.75)

**Table 2:** Prevalence of Suicidal ideation among the Chinese Elderly.

	Overall		Urban		Rural	
Variable	%	*p*	%	*p*	%	*p*
**Overall**	2.77		1.95		3.60	
**Gender**		0.004		0.066		0.006
Male	2.44		1.68		3.12	
Female	3.13		2.21		4.18	
**Age**	-	0.000	-	0.000	-	0.000
**Education level**		0.000		0.000		0.000
Illiteracy	4.67		4.04		4.90	
Primary school	2.42		2.01		2.74	
Junior high school or above	1.48		1.95		2.22	
**CCP membership**		0.000		0.004		0.002
Yes	1.54		1.35		2.03	
No	3.13		2.24		3.82	
**Financial situation**		0.000		0.000		0.000
Sufficient	0.93		0.00		2.17	
Relatively sufficient	0.93		0.94		0.91	
Roughly enough	1.33		1.08		1.64	
Relatively insufficient	5.30		4.31		5.87	
Insufficient	10.12		9.01		10.73	
**Marital status**		0.000		0.002		0.000
In marriage	2.27		1.66		2.96	
Not in marriage	3.74		2.62		4.65	
**Number of children**		0.000		0.001		0.024
**Chronic diseases**		0.000		0.000		0.000
Yes	3.32		2.25		4.55	
No	0.93		0.59		1.16	
**Self-rated health status**		0.000		0.000		0.000
Very unhealthy	11.92		13.07		11.20	
Unhealthy	6.01		4.32		7.14	
Average	1.51		1.09		1.99	
Healthy	0.96		0.70		1.29	
Very healthy	0.72		0.57		0.88	
**Disability of daily living**		0.000		0.000		0.000
**Depression**		0.000		0.000		0.000
**Visiting neighbors**		0.000		0.000		0.000
Frequently	2.24		1.88		2.46	
Occasionally	2.21		1.27		3.15	
Never	4.33		2.94		6.88	
**People who can help you**		0.000		0.000		0.000
Yes	2.41		1.72		3.10	
No	6.19		4.00		8.74	
**Negative life events**		0.000		0.000		0.000
Yes	4.30		3.68		4.78	
No	1.89		1.14		2.77	
**Religious belief**		0.000		0.080		0.000
Yes	3.94		2.55		5.54	
No	2.58		1.85		3.31	

**Table 3:** Logistic Regression of the prevalence of Suicidal Ideation.

	Model 1-Overall		Model 2-Urban		Model 3-Rural	
Variable	OR	*p*	OR	*p*	OR	*p*
**Gender** (Male)	1.055	0.619	1.113	0.566	1.019	0.887
**Age**	1.002	0.811	1.026	0.046	0.990	0.276
**Education level** (Ref. = junior high school or above)						
Primary school	0.863	0.315	0.877	0.516	0.869	0.546
Illiteracy	1.057	0.728	1.049	0.842	1.070	0.775
**CCP membership** (Yes)	1.032	0.837	1.212	0.363	0.817	0.388
**Financial situation** (Ref. = insufficient)						
Relatively insufficient	0.921	0.522	0.922	0.732	0.908	0.535
Roughly enough	0.501	0.000	0.546	0.018	0.466	0.000
Relatively sufficient	0.495	0.009	0.620	0.220	0.371	0.012
Sufficient	0.419	0.235	0.000	0.996	0.677	0.602
**Marital status** (Yes)	1.195	0.102	1.431	0.062	1.056	0.686
**Number of children**	1.073	0.010	1.048	0.371	1.085	0.012
**Chronic diseases** (Yes)	2.000	0.000	1.650	0.154	2.158	0.000
**Self-rated health status** (Ref. = very healthy)						
Healthy	1.037	0.940	0.756	0.715	1.228	0.745
Average	0.777	0.589	0.570	0.447	0.932	0.907
Unhealthy	1.143	0.778	0.807	0.777	1.380	0.597
Very unhealthy	1.167	0.753	1.278	0.754	1.110	0.869
**Disability of daily living**	1.602	0.000	1.305	0.140	1.726	0.000
**Depression**	1.302	0.000	1.338	0.000	1.280	0.000
**Visiting neighbors** (Ref. = never)						
Occasionally	0.666	0.000	0.648	0.019	0.650	0.002
Frequently	0.930	0.591	1.352	0.174	0.767	0.118
**People who can help you**	0.550	0.000	0.559	0.004	0.555	0.000
**Influence of major events**	1.095	0.362	1.412	0.045	0.952	0.692
**Religious belief**	1.462	0.002	1.118	0.588	1.708	0.000
**Location** (Rural)	1.084	0.471				
Constant	0.002	0.000	0.000	0.000	0.005	0.000
Nagelkerke R^2^	0.2282		0.2557		0.2400	
